# Ultrathin Six-Band Polarization-Insensitive Perfect Metamaterial Absorber Based on a Cross-Cave Patch Resonator for Terahertz Waves

**DOI:** 10.3390/ma10060591

**Published:** 2017-05-28

**Authors:** Yong Zhi Cheng, Mu Lin Huang, Hao Ran Chen, Zhen Zhong Guo, Xue Song Mao, Rong Zhou Gong

**Affiliations:** 1 School of Information Science and Engineering, Wuhan University of Science and Technology, Wuhan 430081, China; hml_q1@163.com (M.L.H.); 18627140931@163.com (H.R.C.); xsmao@wust.edu.cn (X.S.M.); 2 Hubei Province Key Laboratory of Occupational Hazard Identification and Control, Wuhan University of Science and Technology, Wuhan 430081, China; zhongbujueqi@hotmail.com; 3 School of Optical and Electronic Information, Huazhong University of Science and Technology, Wuhan 430074, China; rzhgong@hust.edu.cn

**Keywords:** perfect metamaterial absorber, terahertz, six-band, cave-cross patch resonator

## Abstract

A simple design of an ultrathin six-band polarization-insensitive terahertz perfect metamaterial absorber (PMMA), composed of a metal cross-cave patch resonator (CCPR) placed over a ground plane, was proposed and investigated numerically. The numerical simulation results demonstrate that the average absorption peaks are up to 95% at six resonance frequencies. Owing to the ultra-narrow band resonance absorption of the structure, the designed PMMA also exhibits a higher Q factor (>65). In addition, the absorption properties can be kept stable for both normal incident transverse magnetic (TM) and transverse electric (TE) waves. The physical mechanism behind the observed high-level absorption is illustrated by the electric and power loss density distributions. The perfect absorption originates mainly from the higher-order multipolar plasmon resonance of the structure, which differs sharply from most previous studies of PMMAs. Furthermore, the resonance absorption properties of the PMMA can be modified and adjusted easily by varying the geometric parameters of the unit cell.

## 1. Introduction

Since the perfect metamaterial absorber (PMMA) concept was first proposed and demonstrated experimentally by Landy et al. [1], it has become a hot research topic of science and technology. PMMAs have recently been rapidly developed in a wide electromagnetic (EM) spectrum range from microwave [1,2,3], terahertz [4,5,6,7,8,9,10,11], and infrared [12,13,14,15,16] to the visible region [17,18,19,20]. PMMA is not limited by the quarter-wavelength thickness and is also scaled to different EM spectrum ranges due to its geometric scalability. PMMAs have been proposed and demonstrated across a wide range of the EM spectrum and hold great potential for applications such as thermal imaging [12], sensors [16,21,22,23], solar cells [24], thermal emitters [25], and so on. The typical PMMA consists of three functioned layers: a patterned metallic structure (e.g., split ring, cut wire, patch, ring, and so on) as the EM resonator; a dielectric or magnetic substrate as a middle spacer; and a continuous metal film or metal wire as the ground layer. Generally, the PMMA can achieve near-unity absorption based on the fundamental resonance of the EM resonator. By adjusting the shape, size, thickness, and properties of the patterned metallic structure and of the dielectric spacer of the PMMA, the permittivity *ε_eff_*(*ω*) and permeability *µ_eff_*(*ω*) can be equivalent, and thus an impedance can be matched to free space [1,2,3,4,5,6,7,8,9,26]. For this fundamental EM resonance, the electric response stems from the excitation of the electric resonators by the electric field [5,6,7]. The magnetic response is usually provided by pairing the top layer with a metal ground plane or metal wire for an external magnetic field. The strong local EM resonance usually restricts the unique responses to only a single narrow-band absorption, which greatly affects its applications, particularly for biological sensing, thermal imaging, and spectroscopic detection. Thus, simple and effective designs of high-performance multi-band PMMAs are also necessary.

Many efforts have been made to try to achieve a multi-band or broadband high-level absorption for EM waves [27,28,29,30,31,32,33]. Generally, there are two design strategies to achieve multi-band absorption or to extend the absorption bandwidth for PMMAs. One approach is to combine multiple sub-units within a coplanar super-unit resonant structure [6,7,8], and another method is to construct an alternating multiple patterned metallic structure and dielectric layers with different geometric parameters stacked vertically [28,29,30,31,32]. However, both design strategies for the multi-band or broadband PMMAs have some disadvantages: Firstly, the super-unit resonant structure could be very complicated, thus increasing the fabrication cost of the PMMAs. Secondly, there are many interactions between the sub-units resulting in an increased angular dependence in practice. Nearly all of the above-mentioned designs are based on the overlapping of the fundamental resonance of the patterned metallic structure with different geometric parameters and usually neglect the high-order EM response. In effect, high-order resonances of (metamaterials) MMs are vital, but often overlooked in the design of PMMAs. It is very useful to design a multi-band PMMA by combining the fundamental and high-order resonance modes in a single patterned metallic structure [3,8,11,34,35,36,37]. For example, Mao et al. demonstrate a multi-band PMMA based on ancient Chinese coin-shaped structures [3], attributed to the combination of the fundamental resonance (*LC* resonances) and dipole resonances. Dayal reported a multi-band PMMA comprised of metallic circular gold micro-disks separated from a thin metallic film by a dielectric zinc sulphide film, the perfect multi-band absorption originating from the excitation of multi-pole resonances at infrared wavelengths [34]. Dung et al. presented a broad PMMA and clarified that the mechanism of dual-band absorption is due to fundamental and third-order magnetic resonances [35]. Wang et al. proposed a PMMA based on a single patched structure, which can achieve a dual-band and triple-band absorption originating from the fundamental resonance and high-order responses by appropriate geometric parameters designs [36]. However, most designs focus on the dual-band and triple-band PMMAs, some of which are polarization-sensitive, and the multi-band, especially the six-band, PMMAs are rarely proposed and demonstrated.

In this paper, we present a simple and effective design of an ultrathin six-band polarization-insensitive PMMA in the terahertz region. Our design consists of an array of a cross-cave patch resonator (CCPR) and a copper ground plane separated by a thin lossy Gallium Arsenide (GaAs) dielectric film. Six ultra-narrow absorption bands are obtained, and their resonance peaks are on average larger than 95%. Compared with the previous reported PMMAs [3,4,5,6,7,8,9,10,11,29,30,31,32,34,35,36,37,38,39], our design has some advantages: Firstly, our PMMA has a compact unit size design and novel resonance mechanism. Secondly, the simple design of the PMMA has more absorption peaks in a single patterned metallic structure and is also polarization-insensitive for normal incident waves. Thirdly, the Q factors of our design are much larger than those of previous PMMAs. Such a simple and effective design may provide some potential applications in biological sensing, material detection, thermal imaging, and communications at terahertz regions.

## 2. Structure Design and Simulation

**Figure 1:**
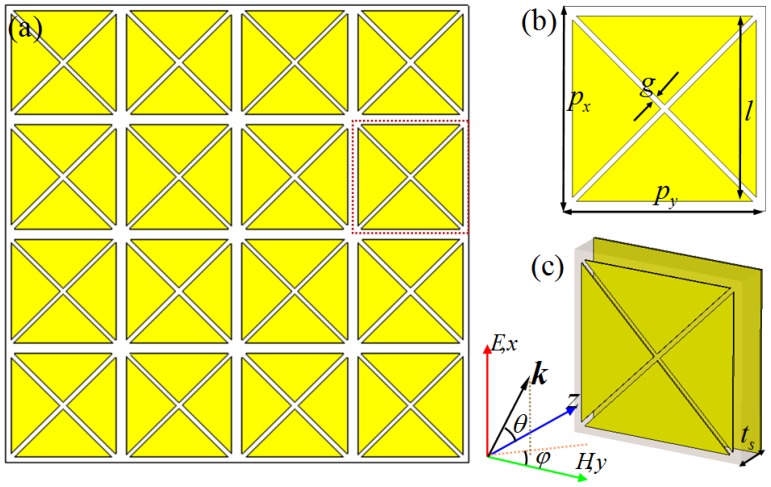
Schematic of the designed six-band polarization-insensitive terahertz perfect metamaterial absorber (PMMA): (**a**) 2D array, (**b**,**c**) front view and perspective view of the unit cell.

We introduce a simple and compact unit cell for a six-band PMMA, as shown in Figure 1. The designed PMMA is composed of a metallic CCPR array over a ground plane layer separated by a dielectric substrate. Figure 1a shows a 2D array structure of the designed PMMA, and the front view and perspective view of the unit–cell structure are displayed in Figure 1b,c. The optimized geometrical parameters of the unit–cell of the PMMA are as follows: *p_x_* = *p_y_* = 75 μm, *l* = 68 μm, *g* = 1 μm, *t*_s_ = 3.8 μm. The unit–cell structure of the PMMA is periodic along the *x* and *y* axes, with periods of 75 μm to avoid diffraction at the normal incidence for frequencies up to 4 THz. In our interesting frequency range (0.8–3.2 THz), the metal elements (CCPR structure and ground plane layer) are made of a lossy copper film with a frequency-independent conductivity σ = 5.8 × 10^7^ S/m and a thickness of 0.6 μm, which is much larger than the typical skin depth in the terahertz regime (to avoid transmission through the ground plane metallic film). GaAs with a complex dielectric constant of *ε* = 12.9 + 0.0774*i* was selected as the dielectric spacer between two metallic layers.

To verify the efficiency and investigate the resonant absorption behavior of our design, the full-wave EM simulations were performed using a frequency domain solver based on finite integration technology (FIT) in a Computer Simulation Technology (CST) Microwave Studio. In the simulation, the periodic boundary conditions in the *x*- and *y*-directions are applied for the transverse boundaries to replicate an infinite array of the PMMA, and the perfectly matched layers are applied along the *z*-direction. The incident electric field and wave vector direction are shown in Figure 1, the periodic array structures being illuminated by a normally incident terahertz plane wave with the electric field parallel to the *x*-axis and the magnetic field parallel to the *y*-axis. The absorbance of the designed PMMA can be calculated by the formulas A(ω)=1−R(ω)−T(ω), where A(ω), R(ω), and T(ω) are the absorbance, reflectance, and transmittance as functions of the frequency ω, respectively. Regarding the plane EM wave normal incidence for our PMMA, no transmission can be examined, as it is blocked off by the continuous copper film. Thus, T(ω)=0, and only the reflectance needs to be measured in our simulations. The absorbance can achieve unity (A(ω)→1) when the reflection is near zero (R(ω)→0) at resonance frequency.

## 3. Results and Discussion

**Figure 2:**
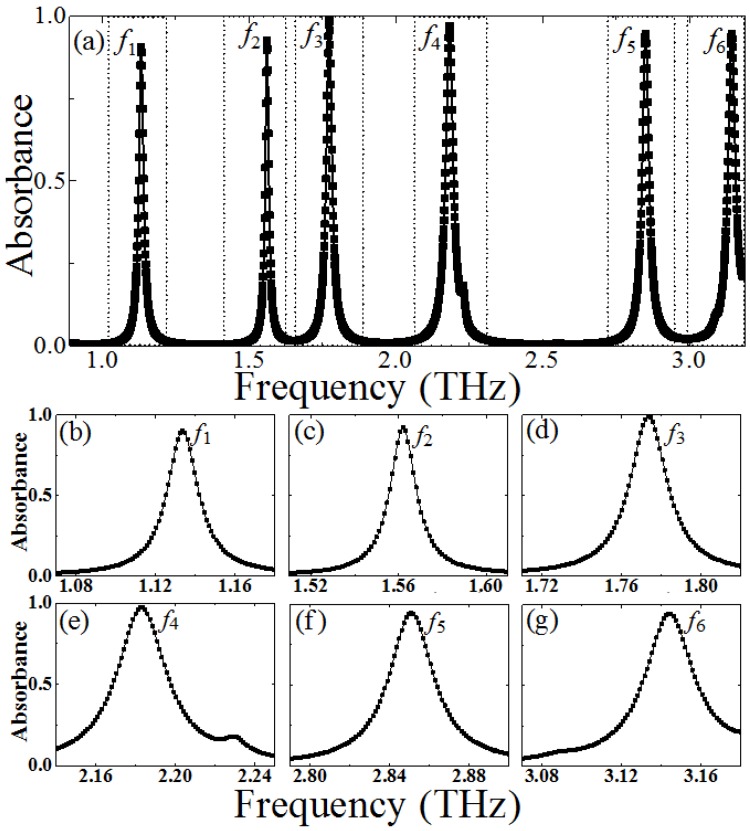
(**a**) The absorption spectra of the proposed six-band PMMA, (**b**–**g**) the absorbance spectra under different resonant frequency domain.

Figure 2 shows the simulated absorbance spectra of the proposed PMMA: six resonant frequencies (*f*_1_, *f*_2_… *f*_6_) can be observed clearly. From Figure 2b–g, at resonant frequencies of *f*_1_ = 1.13 THz, *f*_2_ = 1.56 THz, *f*_3_ = 1.77 THz, *f*_4_ = 2.18 THz, *f*_5_ = 2.85 THz, and *f*_6_ = 3.14 THz, the absorbance *A(ω*) is about 90.5%, 94.4%, 98.7%, 96.2%, 95.4%, and 95.2%, respectively. The corresponding electric thickness of the PMMA is about λ_1_/70, λ_2_/50, λ_3_/45, λ_4_/36, λ_5_/28, and λ_6_/25, respectively (the λ*_i_* is the resonance wavelength, where *i* = 1, 2, 3…6). Thus, our designed PMMA possesses an ultrathin thickness compared with the operation wavelength (<λ/25, at 3.14 THz). In addition, it also exhibited a frequency selectivity of the six-band PMMA, since the bandwidth of perfect absorption is very narrow and the off-resonance absorption is very small (*A*(*ω*) < 5%). The peak absorption at different resonant frequencies corresponds to the nature of the different resonance modes, which will be illustrated and classified by analyzing the distributions of the electric fields of the unit–cell structure. It can be conjectured that the high-level absorption of those six resonance peaks is attributable to the higher-order multipolar plasmon resonances of the CCPR structure. It can be found that the absorption frequency band for the six-peak PMMA is relatively narrow compared with the previous PMMAs [5,6,7,8,11,12,36,37]. It is expected that the proposed PMMA has a significantly higher Q factor than the previous ones.

The Q factor is usually defined as the ratio of the central frequency to the full width at half maximum (FWHM) bandwidth of the resonance, and this was calculated for our design. At the above six resonant frequencies (*f*_1_ = 1.13 THz, *f*_2_ = 1.57 THz, *f*_3_ = 1.77 THz, *f*_4_ = 2.18 THz, *f*_5_ = 2.83 THz, and *f*_6_ = 3.14 THz), the FWHM bandwidth is about 0.0167 THz, 0.0139 THz, 0.0219 THz, 0.0219 THz, 0.0251 THz, and 0.0286 THz, respectively. Thus, the corresponding Q factor is about *Q*_1_ = 67.48, *Q*_2_ = 113.19, *Q*_3_ = 80.6, *Q*_4_ = 77.39, *Q*_5_ = 112.67, and *Q*_6_ = 109.53, respectively. From the above results, the high-level absorption with high Q factor only occurs at resonant frequencies. The Q factor of the previous MMs structure for sensing applications is usually relatively lower (Q factor < 20) [21,22,23,40], in contrast, our proposed PMMA has a relatively higher Q factor (>60). Especially, it can be expected that our proposed PMMA can serve as a highly sensitive sensor for phase imaging of prohibited drugs, detection of combustible, toxic and harmful gases, and biological sensing, due to its high Q factor. In addition, it can be expected that the proposed structure of the PMMA is insensitive to the polarization state of the incident terahertz wave, due to the high geometric rotational symmetric of the unit–cell structure.

**Figure 3:**
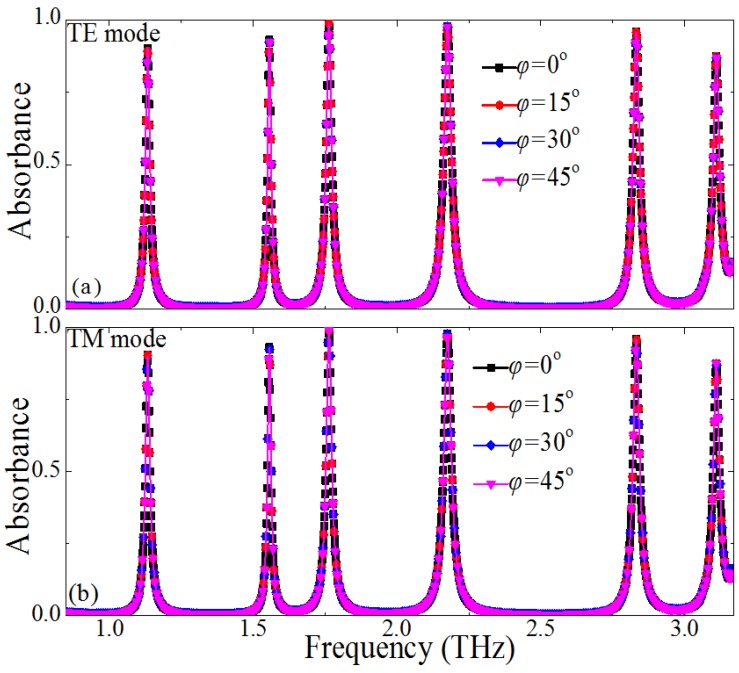
Dependence of the absorption spectra on the polarization angles of the normal incident terahertz wave for the proposed PMMA: (**a**) transverse electric (TE) mode and (**b**) transverse magnetic (TM) mode.

We characterized the polarization angle dependence of the PMMA for both TE and TM waves under normal incidence, and the results are shown in Figure 3. We only needed to consider the polarization angles from 0^°^ to 45^°^, owing to the rotational symmetry of the unit–cell structure of the PMMA, as shown in Figure 3a,b. Obviously, under normal incidence, the absorbance under different polarization angles remains unchanged for both the transverse electric(TE) and the transverse magnetic (TM) modes. This means that the designed PMMA can keep the absorption stability for normal incident terahertz waves with different polarization in practical application. It should be noticed that the first absorbance can be kept unchanged for both the TE and the TM mode, when the angle of the incident wave is below 65^°^. The absorbance performance of the higher-resonant frequencies (for example, second resonance, third resonance…and sixth resonance frequency) will deteriorate with the increase of the incident angle (*θ* > 30^°^), due to the higher-order multipolar plasmon resonance (not shown).

To illustrate the resonant absorption mechanism of the PMMA, several physical interpretations or theory modes have been proposed and demonstrated, such as the effective media theory for impedance matching [41,42], the electric or the magnetic resonance theory [43,44,45,46], the interference theory [47,48], the coupled mode theory [49], the surface plasmon theory [50], the standing-wave theory [51], and the equivalent *LC* circuit theory [52,53]. When using these theory modes to analyze and explain the underlying mechanism of the proposed PMMAs, they are persuasive and convincing. However, most of these theory modes have some limitations, for example, when using the coupled mode theory, the higher-order resonance modes are usually ignored and not considered. In all of these physical interpretations or theory modes, the electric or magnetic resonance mechanisms are closer to the physical nature of the MMs structure. Thus, in this work, we elucidate the underlying physics mechanism of the multi-band PMMAs by observing and analyzing the resonant response of the CCPR structure for normal incident THz waves. Similarly to previous works [54,55], we only simulated electric field and power loss density distributions of the unit–cell structure to analyze the physics mechanism of our proposed PMMA.

**Figure 4:**
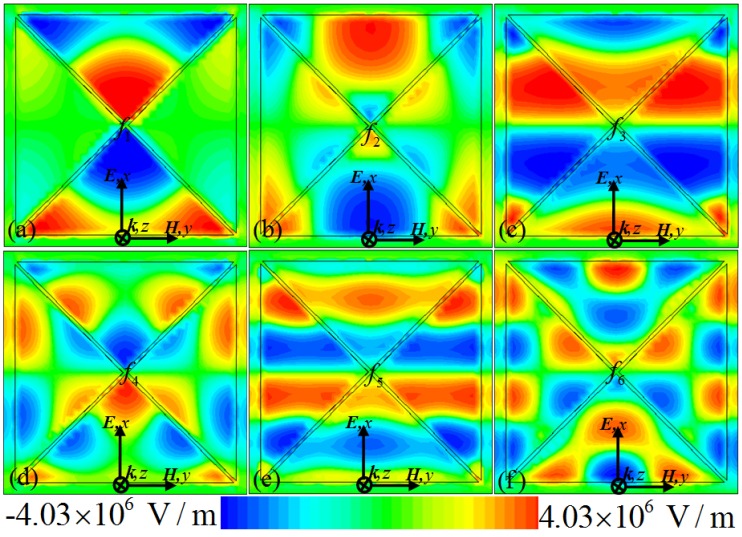
Distributions of the *z*-component (*E_z_*) of the electric field for the proposed PMMA at frequencies of (**a**) *f*_1_ = 1.13 THz, (**b**) *f*_2_ = 1.56 THz, (**c**) *f*_3_ = 1.77 THz, (**d**) *f*_4_ = 2.18 THz, (**e**) *f*_5_ = 2.85 THz, and (**f**) *f*_6_ = 3.14 THz, respectively.

Figure 4 shows the simulated electric field distributions at the different resonant frequencies, which can provide insight on the physical nature of the resonance absorption of our proposed PMMA. It can be observed that the *z*-component (*E_z_*) of the electric field of the incident wave is mainly concentrated on the patch edges, gap edges, and corners of the metallic CCPR structure. As shown in Figure 4a, at the lowest frequency (*f*_1_ = 1.13 THz) the electric field is mainly concentrated on the corners of the upper and lower triangle areas of the CCPR structure, indicating an excitation of quadrupolar resonance. This means that the upper and lower triangle areas of the resonator structure can strongly couple with the electric field and supply quadrupolar resonances, which can be interpreted by a simple dipole–dipole interaction along the electric field direction [38,39,43,44]. For the second frequency (*f*_2_ = 1.56 THz), as shown in Figure 4b, the upper and lower areas of the CCPR structure and the greater part of the triangle section generate the half-wave resonance mode, coupling strongly to the electric field. Similarly to the lowest mode (*f*_1_), the CCPR structure at the second mode (*f*_2_) supplies hexapolar resonance. In effect, the electric field distributions revealing quadrupolar and hexapolar resonances correspond to the nature of localized surface plasmon (LSP) behaviors [56,57]. Figure 4c shows that for the third resonant frequency (*f*_3_ = 1.77 THz) the electric field (*E_z_*) distribution is mainly concentrated on the upper, middle and lower areas of the CCPR structure, showing an excitation of multiple half-wavelength charge oscillations in the structure corresponding to the first higher-order mode [34]. Essentially, the higher-order modes occurring at the higher frequencies are due to the fact that the dimension of the CCPR structure is larger than the multiple of a half-wavelength of the resonant modes [8,11,34,36,39]. Similarly, as shown in Figure 4e, the *E_z_* distribution at the fifth frequency (*f*_5_ = 2.85 THz) reveals the next higher-order excitation of multiple half-wavelength charge oscillations in the CCPR structure. The *E_z_* distributions for the higher-order mode possesses a finite dipole moment for these two modes (*f*_3_ and *f*_5_), which is much like the fundamental dipole resonance response [30]. At the other frequencies (*f*_4_ = 2.18 THz and *f*_6_ = 3.14 THz), as shown in Figure 4d,f, the *E_z_* distributions reveal decapole and octadecapole excitations of the CCPR structure [57]. It can be seen that the resonant electric fields associated with the multipolar modes (*f*_4_ and *f*_6_) are highly localized on the CCPR structure as well as highly enhanced in comparison to fields at nearby frequencies. It should be noted that the excitations of the propagating surface plasmon (PSP) also contribute to the formation of the absorption peaks (*f*_4_ and *f*_6_) [57]. This also means that the fourth and sixth absorption peaks (*f*_4_ and *f*_6_) originate from the combination of the high-order LSP and PSP resonance of the designed CCPR structure [57]. Therefore, this six-band perfect absorption of the PMMA is realized easily, based on the combination of the PSP resonance and the high-order multipolar response of the CCPR structure. These results suggest a new approach for designing a multi-band PMMA by integrating different resonance modes in a single patterned structure.

**Figure 5:**
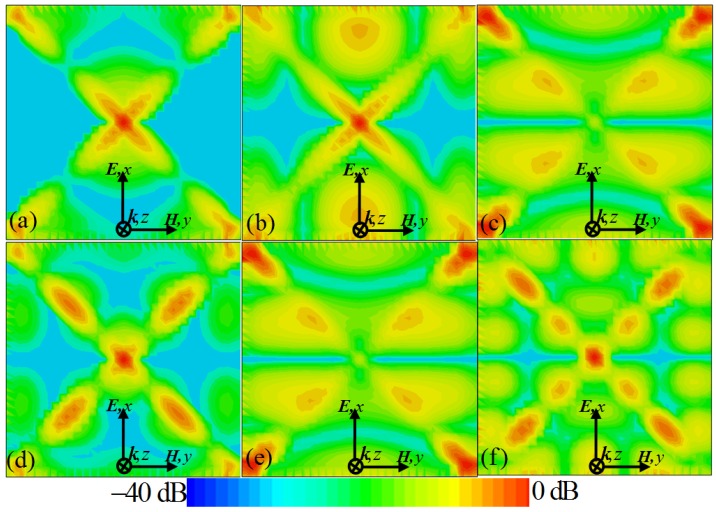
Distributions of power loss density in the middle dielectric layer for the proposed PMMA at frequencies of (**a**) *f*_1_ = 1.13 THz, (**b**) *f*_2_ = 1.56 THz, (**c**) *f*_3_ = 1.77 THz, (**d**) *f*_4_ = 2.18 THz, (**e**) *f*_5_ = 2.85 THz, and (**f**) *f*_6_ = 3.14 THz, respectively.

To further characterize the terahertz wave resonance absorption behavior of the proposed six-band PMMA, we provided the distributions of the power loss density of the unit–cell structure at different resonant frequencies, as shown in Figure 5a–f. It can be clearly observed that the regions of maximum power losses occur mainly around the gap, the upper and lower edges, and other side areas of the middle dielectric layer for the proposed PMMA. In effect, the majority of the terahertz wave EM energy is dissipated as dielectric loss in the middle dielectric layer at the different resonance modes. For example, as shown in Figure 5a, the distribution of power losses is mainly concentered on the gap of the structure, which is induced by the excitation of quadrupolar resonance. From the Figure 5b–f it can be observed that the properties of the power loss density distributions are similar to those of the electric field distributions. Obviously, the distributions of power loss density associated with the higher-order multipolar modes are highly localized for the CCPR structure and the middle dielectric layer at the different resonance modes. Thus, it can be concluded that the PSP resonance and high-order multipolar resonances play an important role for the high-level absorption at the resonant frequencies. 

Based on the above explanations of the resonance absorption mechanism of the six-band PMMA, the influence of geometric parameters of the unit–cell structure on the resonance frequencies could be easily understood. It can be conjectured that the resonance absorption frequencies of the PMMA mainly depend on the length *l*, gap width *g* of the metallic CCPR, and the thickness *t_s_* of the dielectric layer. Taking a further step, we studied the influences of geometric parameters of the unit–cell structure on the resonance absorption properties of the proposed PMMA.

**Figure 6:**
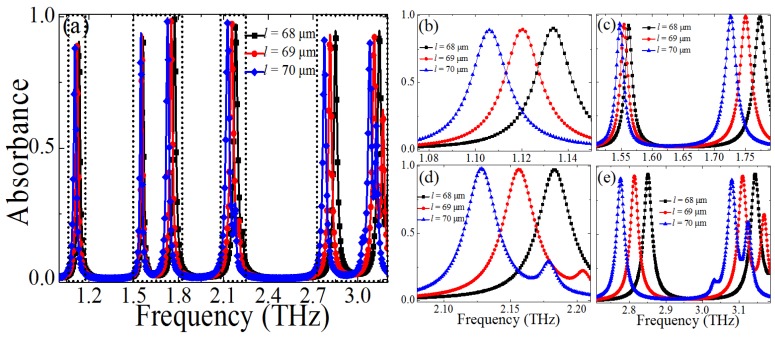
(**a**) Dependence of the absorption spectra of the proposed PMMA on the size changes of the cross-cave patch resonator (CCPR) length *l* (*l* = 68 μm, 69 μm, 70 μm), (**b**–**e**) dependence of the absorbance spectra on the *l* at different frequency domains.

Firstly, the PMMAs with different CCPR lengths *l* (*l* = 68 μm, 69 μm, 70 μm) were calculated when the other geometric parameters were fixed, as shown in Figure 6. It can be observed that the CCPR length *l* can influence the all resonant frequencies (*f*_1_, *f*_2_, *f*_3_, *f*_4_, *f*_5_, and *f*_6_), which will decrease with the increase of the *l*. The absorption peaks of the resonance modes *f*_1_ and *f*_3_ will remain almost unchanged, and those of the *f*_2_ and *f*_4_ will increase slightly, while the ones of the other resonances (*f*_5_ and *f*_6_) will decrease slightly with the increase of the *l*. In addition, it should be noted that another peak close to *f*_6_ can be observed clearly when the CCPR length is greater than 68 μm (>68 μm), revealing that the higher-order resonance mode is excited in this case. However, the absorbance of the resonant frequency close to *f*_6_ is relatively small (<70%). According to the equivalent *LC* resonance circuit theory, the resonant frequency can be expressed as $f_{i}=\frac{1}{2\pi \sqrt{LC}}$, where the equivalent capacitance *C* and inductance *L* are mainly determined by the geometric parameters (*l*, *g*, and *t_s_*) of the unit–cell structure of the PMMA [51,52,58]. The *C* will increase with the increase of the *l*, thus resulting in a decrease of the multiple resonant frequencies. 

**Figure 7:**
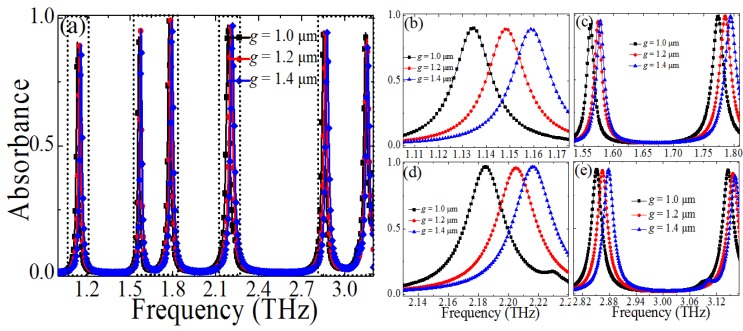
(**a**) Dependence of the absorption spectra of the proposed PMMA on the size changes of the CCPR gap width *g* (*g* = 1 μm, 1.2 μm, 1.4 μm), (**b**–**e**) dependence of the absorbance spectra on the *g* at different frequency domain.

Next, we discuss the effect of the CCPR gap width *g* on the absorption, and the absorbance of the PMMAs with different *g* (*g* = 1 μm, 1.2 μm, 1.4 μm) was calculated when the other geometric parameters were unchanged, as shown in Figure 7. From Figure 7b–e it is seen that the resonant frequencies (*f*_1_, *f*_2_, *f*_3_, *f*_4_, *f*_5_, and *f*_6_) drift to the higher frequency, and the absorption peaks remain almost unchanged when the parameter *g* was changed from 1 μm to 1.4 μm. Although the resonance modes (*f*_3_ and *f*_6_) also shift to the higher frequency, the absorption peak of mode *f*_6_ will decrease with the increase of the CCPR gap width *g*. It also can be easily understood that *C* will decrease with the increase of the CCPR gap width *g*, thus resulting in an increase of multiple resonant frequencies, which is different to the change of the *l*.

**Figure 8:**
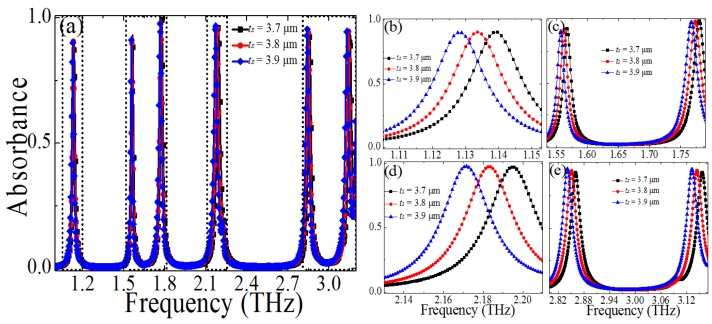
(**a**) Dependence of the absorption spectra of the proposed PMMA on the size changes of the dielectric layer thickness *t_s_* (*t_s_* = 3.7 μm, 3.8 μm, 3.9 μm), (**b**–**e**) dependence of the absorbance spectra on the *t_s_* at different frequency domain.

Furthermore, we discuss the effect of the dielectric layer thickness *t_s_* on the absorption, and Figure 8 shows the calculated absorbance of the PMMA with different *t_s_* (*t_s_* = 3.7 μm, 3.8 μm, 3.9 μm) while the other geometric parameters were unchanged. From Figure 8b–d, it is obvious that the resonance absorption frequencies (*f*_1_, *f*_2_, *f*_3_, and *f*_4_) drift to the lower frequency, and the absorption peak remains unchanged at a high level when changing the parameter *t_s_* from 3.7 μm to 3.9 μm. Although the absorption frequencies (*f*_5_ and *f*_6_) also shift to the higher frequency, the absorption peaks will increase with the increase of the dielectric layer thickness *t_s_*, as shown in Figure 8e. In this case, when increasing the dielectric layer thickness *t_s_*, the *L* will increase, thus the multiple resonant frequencies will decrease accordingly.

Based on the above calculation and analysis, the absorption peaks and frequencies are sensitive to the geometric parameters (*l*, *g*, and *t_s_*) of the unit–cell structure. We could adjust the absorption peaks and frequencies by changing these parameters. Although all changes of the parameters almost affect the resonant frequency absorption peak, the designed PMMA still remains high absorption level (*A(ω*) > 90%) at resonance. These results further confirm that the frequencies of the designed six-band PMMA could meet different application needs, especially in sensors.

## 4. Conclusions

In summary, we present an ultrathin six-peak PMMA based on a metallic square cross-cave patch structure placed over a ground plane separated by a dielectric substrate. Simulations confirm that the absorption peak of the PMMA is on average more than 95% at six different resonant frequencies. The designed PMMA exhibits a higher Q factor of more than 65, due to the ultra-narrow band resonance absorption of the structure. Thus, it can be expected that our proposed PMMA can be applied in spectroscopic detection and biological sensing, due to its higher Q factor. Moreover, the high absorption level of the designed PMMA can be kept almost unchanged with different polarization angles for both TE and TM waves under normal incidence. The absorption mechanism of this design was illustrated by studying the electric field distributions at six resonant frequencies. The electric field distributions for different frequencies (*f*_1_, *f*_2_…*f*_6_) revealed that the high-level absorption originated from the PSP and the higher-order multipolar plasmon resonance response of the square cross-cave patch structure. Furthermore, the resonance absorption properties of our design can be adjusted by varying the geometric parameters of the unit–cell structure, which gives considerable freedom to shift or change the operation frequencies of the PMMA to meet different application needs. In addition, the simple design of the six-band PMMA is easily fabricated using the conventional photolithography process and metallization process steps [59,60]. In our next work, we will perform an experiment for our designed PMMA for practical sensing application. The aforementioned advantages of the six-band PMMA make it a good candidate in some potential applications of thermal imaging, wavelength selective radiators, thermal bolometers, biosensors, and so on.
